# Horticultural Therapy for Improving the Work Performance and Interpersonal Relationships of Persons with Intellectual Disabilities

**DOI:** 10.3390/ijerph192113874

**Published:** 2022-10-25

**Authors:** Hyo-Jung Son, Dae-Sik Kim, Sin-Ae Park

**Affiliations:** 1 Department of Bio and Healing Convergence, Graduate School, Konkuk University, Seoul 05029, Korea; 2 Department of Local Environment Engineering, College of Agriculture & Life Sciences, Chungnam National University, 99 Daehak-ro, Yuseong-gu, Daejeon 34134, Korea; 3 Department of Systems Biotechnology, Konkuk University, Seoul 05029, Korea

**Keywords:** intellectual disability, vocational rehabilitation, upper limb function, horticultural therapy, gardening

## Abstract

For the occupational adaptation and social integration of the intellectually disabled, it is helpful to improve their work performance and interpersonal skills. The purpose of the study was to evaluate the effectiveness of horticultural therapy (HT) programs to improve work performance and interpersonal relationships of persons with intellectual disabilities. Based on observations and analyses of how people with intellectual disabilities work, we have developed a 12-session HT program that includes upper limb movements and physical activities to improve hand function. We recruited, with the consent of their legal guardians, 14 (6 males, 8 females) participants who had intellectual disabilities and were working at a sheltered workshop in K-gu, Seoul, South Korea. The program consisted of twelve sixty-minute sessions that were conducted twice a week at a rooftop garden. For pre- and post-evaluation of the program, the survey of functional adaptive behavior (SFAB), interpersonal negotiation strategies, a horticultural job evaluation (self), hand function tests (pegboard, pinch gauge, fingertips), and blood sample tests for physiological indicators of exercise were conducted. Interpersonal negotiation strategies, functional adaptive behaviors, and physical abilities for job behaviors, including agility and grasping of the hand, improved significantly from before to after the program (*p* < 0.05). A positive result of VEGF (vascular endothermic growth factor) in blood sample tests implies the need for further research on cognitive changes caused by horticultural activities. This study has limitations due to the small number of participants, but the results suggest that low- to medium-intensity horticultural treatment programs using the upper body and hands could be effective for vocational rehabilitation of the intellectually disabled.

## 1. Introduction

The AAIDD (2021) defines “intellectual disability” as, “a condition characterized by significant limitations in both intellectual functioning and adaptive behavior that originates before the age of 22”. It also defines adaptive behavior as, “the collection of conceptual, social, and practical skills that are learned and performed by people in their everyday lives”. The number of persons in South Korea with intellectual disabilities has increased steadily from 2015 to 2021: 189,752 in 2015; 200,903 in 2017; 212,936 in 2019; and 221,557 in 2021, increasing by 2–3% every year [1]. As members of society, individuals with disabilities also have jobs and maintain a stable work–life balance to achieve quality of life, productivity, subjective well-being, and social integration within their communities [2].

An occupation relieves the symptoms of disability and helps people with intellectual disabilities to maintain activities of daily living [3,4]. Through their jobs, people gain economic stability and interact with others to establish and maintain relationships [5]. However, society has socially rejected people with disabilities because of their lack of academic achievement, failure to fit into society, and limitations in interpersonal, family, and social relationships. Thus, they may feel frustrated, angry, or insecure, and in some cases, they may become violent [6]. These characteristics have lowered their employment rate, and even if they are employed, they have trouble adapting [7]. Therefore, job performance that supports job adaptation is essential for people with intellectual disabilities who are either already employed or preparing for employment. Vocational rehabilitation involves a series of goal-oriented, individualized, continuous processes that are systematically planned to help people with disabilities in adapting to work as a profession through the proper involvement of rehabilitation specialists, who facilitate the development of their potential to maximize their remaining functions and integrate them into society to serve the interests of citizens [8]. Although persons with intellectual disabilities have limitations in their abilities, the application of occupational maintenance factors through proper environmental interventions can improve their functionality and help them maintain their jobs.

Horticultural therapy (HT) is defined as a complementary and alternative treatment in which a professional therapist uses horticultural activities to treat clients with special needs [9]. In studies on the physical effects of HT, most horticultural activities were conducted outdoors and contributed to the development of motor effects, muscle functions, increased flexibility, endurance, and concentration [10,11,12]. In addition, several studies have investigated the physiological healing mechanisms of horticultural activities by exploring their potential as aerobic exercise with high energy consumption at a low to high intensity, and as muscular exercise that use whole body muscles [13,14,15,16,17]. Arranging flowers can simultaneously induce various upper limb movements and functions in the sitting position while performing various tasks such as cutting, curling, winding, placing, and rolling [18]. Park et al. [19] suggested that vocational rehabilitation with HT was effective in enhancing vocational rehabilitation services because it improved the movement of small muscles and reduced inferiority in task performance. We classified horticultural activities into upper limb movements (planting and sowing) and lower limb movements (digging, raking, planting, sowing, harvesting, and weeding) based on a study by Lee [20], who improved hand functions through the kinematic and kinetic analysis of HT in stroke patients and applied the findings into a program. We developed a 12-session HT program consisting of indoor and outdoor horticultural activities that included upper and lower physical activities with low- (2–3 Mets) to moderate (3–5 Mets)-intensive exercises [14,17].

Since HT facilitates sharing and working together by offering opportunities for interaction, it can also improve the communication and social skills of persons with intellectual disabilities. In a previous study, an HT training program that was used in a facility for vocational rehabilitation of persons with intellectual disabilities improved the persons’ social, psychological, emotional, and work performance, thus showing its appropriateness as a job preparation program for those with intellectual disabilities [21].

Cognitive factors such as attention and concentration are important for intellectually disabled people to successfully perform their tasks in the information society [22]. Previous studies reported that gardening can be considered aerobic and muscular exercise, and explained that gardening intervention as a physical activity is beneficial to cognitive function and memory. For example, there are reports that a horticultural therapy program (20 sessions) designed with general gardening activities has significantly improved the cognitive function of the elderly with dementia [12]. However, it is rare to measure the level of brain nerve growth factors related to cognitive functions, such as BDNF, VEGF, and IGF-1, by executing a horticultural therapy program for the intellectually disabled. This study examined the effect of HT programs on work performance and interpersonal relationships of intellectually disabled people working in a sheltered workshop, as well as the changes in brain nerve growth factors related to cognitive function.

## 2. Materials and Methods

### 2.1. Research Participants

To recruit participants for this study, we sent flyers that described the purpose and period of the HT program to persons with disabilities working at a sheltered workshop in K-gu, Seoul, South Korea. In total, 14 persons with intellectual disabilities agreed to participate in our study with their legal guardians’ consent. During the orientation session, we presented detailed information about the program and asked participants to complete demographic information forms (Table 1).

The average age of the 14 participants—6 males (43%) and 8 females (57%)—was 33.9 ± 9.4 years, and their average number of working years in the protected workplace was 7.3 ± 4.5 years. Most of the participants were moderate (IQ 35–49) or severely (IQ 20–34) intellectually disabled (Table 1). The attendance rate for the HT program was 100%.

### 2.2. Developing a Horticultural Therapy Program for Improving Work Performance and Interpersonal Relationships of Persons with Intellectual Disabilities

To develop a HT program for improving the work performance of persons with intellectual disabilities, the work performed by the disabled in a sheltered workshop was investigated and analyzed. We observed 20 persons with intellectual disabilities in a sheltered workshop for 2 h and recorded the work content, stages, and major body parts used during their work. Persons in the sheltered workshop with various disabilities were making paper bags, which was divided into five stages, ranging from material preparation to finishing bags. The participants of our program with intellectual disabilities performed the final stage, the process of stitching the finished paper bags, which was further divided into four steps.

The first step was to insert the bag strap into a hole on one side of the paper bag. The second was to remove the bag strap and insert it into a hole on the opposite side. This involved delicate work using the thumb and index fingers and required eye–hand coordination. The third step was to pull the straps on both sides and tie them together, which required grip and power in both hands. The last step was to move the finished bags to the side and arrange them using the hands and arms. These tasks were judged to require hand function and upper limb force.

Through the above work analysis, we developed a 12-session HT program for the intellectually disabled to enhance upper limb movements through seed planting, shoveling, plant planting, etc., improving the work performance and interpersonal relationships in the workshop. To improve the first and second steps of strapping paper bags that required agility and eye–hand coordination, basic horticultural activities such as sowing and cutting were required. In particular, the difficulty and speed of the work were controlled by varying the size of the seeds. In the first stage of sowing (third session), we requested that each large seed, such as sunflower seeds, be placed correctly in a tray.

In the second stage (4th session), three medium-sized radish seeds were planted in a tray. In the third stage (10th session), a program was developed to improve concentration and hand skills by planting small red mustard seeds (*Brassica juncea* [L.] Czern). Regarding the use of both hands, the participants had to hold a wooden chopstick in their left hand to make a hole and place the seeds with their right hand. Cutting was performed in two sessions (5th and 7th). In the 5th session, succulent plants that fell easily, such as Sedeveria Letizia and Crassula, were used. We asked participants to fill the trays with soil and then insert the succulent leaves in a sequence. In the 7th session, to increase the difficulty, stems of herb plants were cut directly with scissors. We chose plants that could be easily cut with scissors, such as apple mint (*Mentha suaveolens*) and geranium (*Pelargonium inquinans* Aiton). The use of hands and arms for trimming the stems and leaves can be linked to the last step of bag making. Since water management was important after planting seeds, we created a “plant care card” to manage water during the 12 sessions and to enable daily physical activity. Participants visited the roof garden daily to conduct activities such as watering, weeding, and talking to plants, and received confirmation from social workers. To manage the sown seeds, seedling sprayers were prepared for them, and they were asked to spray five times with each hand so that both hands could be trained to increase their grip and strength. This task can be linked to the tie-up process using grip power in the third step of bag making. In the 6th and 8th sessions, the sprouted plants were transferred to a garden and harvested. In the 11th session, rooted succulent and herb plants were planted to decorate their pots. In the last session, soap flower baskets were made by inserting soap flowers into baskets at regular intervals to create shapes. Before the activity, the participants practiced placing dots at regular intervals and inserting flowers in the Styrofoam. At first, the process progressed slowly, but after three or more repetitions, accuracy and speed increased. Overall, at the end of the 12-session HT program, persons with intellectual disabilities were able to improve their interpersonal relationships. They learned to be responsible by taking care of their plants and understood how to communicate with other participants. This program was developed by analyzing the steps required for work at sheltered workshops, after considering the characteristics of the persons with intellectual disabilities, and systematically applying the results from this analysis to horticultural activities.

### 2.3. Implementing the HT Program for Persons with Intellectual Disabilities

A 12-session HT program was developed that consisted of sessions twice a week, each lasting 60 min (see Table 2). The sessions were conducted at a rooftop garden installed in a protected workplace. One primary instructor, two assistant instructors, and one social worker participated in the program.

To improve the hand functions, interpersonal relationships, and physical activities of the participants, the activities were subdivided, and language instructions with demonstrations were provided at each session [23]. At each introductory session, we greeted each other, and shared dates and past activities for rapport formation. In addition, we checked the “plant care card” and introduced the garden activities to be performed on that day. Before starting the activity, a 5 min warm-up exercise was performed to loosen the body tensed by indoor work. In the development phase, we divided ourselves into groups and chose our group leader to perform the garden activities. Around 4–5 persons were assigned to a group, and they were able to communicate with each other by planting, managing, and harvesting crops in their group’s garden. Furthermore, activities that used the entire body such as weeding, picking out stones, and fertilizing were performed. Leafy plants of various colors and edible flowers were planted to enable visual stimulation and healing. For watering activities, participants were taught correct posture while gardening, such as stepping on the foot and bending the back to improve their balance and lower limb functions. In the program’s final phase, we mentioned the importance of regular garden management, encouraged them to be responsible for watering activities, and briefly introduced the next session to draw attention to the program. This program was developed to improve participants’ work abilities and interpersonal relationships by increasing their hand functions and overall physical abilities through horticultural activities.

### 2.4. Assessments

For the effectiveness of the HT program, a hand function evaluation, interpersonal bargaining strategy evaluation, and functional adaptation behavior survey (SFAB) were conducted to analyze the work performance and interpersonal relationships of the intellectually disabled. In addition, the level of brain nerve growth factors related to cognitive functions, such as BDNF, VEGF, and IGF-1 through blood, was analyzed. In order to change the brain nerve growth factor related to the cognitive function of the intellectually disabled, 10 mL of blood was collected before and after the program to analyze the change in the brain renal management factor. A horticultural job evaluation and a self-report questionnaire were conducted before and after the program to evaluate horticultural practice skills, responsibilities, and physical management skills. Subsequently, the participants’ satisfaction with the program was evaluated.

The Grooved Pegboard (Motel SH-7446, Preston, CT, USA) is used for neuropsychological, visual–motor coordination, and occupational assessments in both adults and children. We asked participants to randomly insert a key peg into 25 holes. The total time used to complete the task was measured only once for each hand [24]. A hand dynamometer (Model 78010, Lafayette Instrument Company, Lafayette, IN, USA) was used to evaluate the grasp power. A higher value indicated a stronger grip. A Jamar hydraulic pinch gauge (749805, Baseline Lite 50 Lb, USA, Sammons Preston) measured finger strength and pinch ability. With the index finger up and the thumb down, participants pressed the button with their fingertips. A higher figure indicated a stronger finger strength.

The interpersonal negotiation strategies questionnaire, developed by Beardslee, Schultz, Selman et al. (1986) and modified by Kwon (1989), was used. Suo (1999) reported a Cronbach’s α of 0.76. The relationship with others was expressed at the level of 0 to 3 and evaluated on a 5-point Likert scale. A higher final rating score indicated more positive interpersonal relationships.

To assess the SFAB, we partially reconstructed its composition and used the McCarron-Dial System (MDS), an evaluation tool that assesses residential, daily life, learning, and vocational skills. Among all the tests, we only used the vocational skill test. A three-point scale (0: not well; 1: partially well done; 2: well done) was used in this test, which consisted of 20 items. In part A, we measured the agility of the fingers and hands, stamina, lift and move skills, upper body coordination, speed, and full-body coordination as physical abilities. In part B, appearance, courtesy, rule compliance, patience, and productivity were evaluated for work performance.

Based on the horticultural activity performance assessment (HAPA) tool, developed by Kim [25] to evaluate the horticultural activity performance of persons with intellectual disabilities, we made revisions to suit the level of the horticultural work skills of intellectual disabilities. The questionnaire consisted of three subcategories: practical technical competence (horticultural skills/horticultural job cognitive abilities), social competence (completion abilities/other considerations/abilities), and physical and mental health competencies (physical management abilities/stress management abilities). The questions were simple, could be easily understood by persons with disabilities, and participants facilitated the evaluation of their own activities [26].

The HT program investigated the effects of brain nerve growth factors related to cognitive function in intellectually disabled people. A professional nurse collected 10 mL of blood before and after the activity, and analyzed three types of brain nerve growth factors, including BDNF (brain-derived renal management factor), VEGF (vascular endothelial elongation factor), and IGF-1.

### 2.5. Paper Bag Strapping Work Activity Evaluation

Paper bag strapping is a work activity for persons with intellectual disabilities in protective workplaces. To evaluate the improvement of hand functions, 14 persons with intellectual disabilities performed the paper bag strapping activity for 5 min before and after the program. The activity was conducted in the same place under the supervision of the same social worker. The number of completed paper bags per participant was counted.

### 2.6. Data Analysis

The demographic information and satisfaction questionnaire results were analyzed using descriptive statistics in Excel (Microsoft Office 2007; Microsoft Corp., Redmond, WA, USA). The level of statistical significance was set at *p* < 0.05. The Wilcoxon verification of a nonparametric paired t-test was analyzed using SPSS (version 24 for Windows; IBM, Armonk, NY, USA) for the hand function evaluation, SFAB, interpersonal negotiation strategy, and horticultural job evaluation results before and after HT program.

## 3. Results

### 3.1. Hand Function Evaluation

#### 3.1.1. Grooved Pegboard Test (GPT)

For the GPT, the time taken to complete the task with each hand was measured. On the dominant hand, the average completion time decreased from 146.3 s to 121.7 s (*p* = 0.05) (Table 3).

#### 3.1.2. A Gyrometer and Pinch Gauge

A gyrometer and pinch gauge were used to record the highest score among the three results of the dominant hand. The HT program significantly improved grip power from 18.6 to 20.8 (*p* = 0.04). As a result of the pinch gauge, the score significantly increased from 6.5 to 7.4 after the program (*p* = 0.02 (Table 3)).

### 3.2. Bag Strapping Evaluation

To evaluate the effects of the HT program, we conducted a paper bag strapping task for 5 min before and after the program (Table 4). The number of bag completions increased significantly from 9.9 to 11.3 bags after the program (*p* = 0.00).

### 3.3. Interpersonal Negotiation Strategies

The results of the subcategory-wise analysis of interpersonal negotiation strategies are presented in Table 5. After the program, significant increases were found in the means of impulse-physical (level 0), which changed from 14.4 to 15.2 (*p* = 0.02), and unilateral (level 1), which changed from 14.1 to 15.7 (*p* = 0.01). However, there were no significant changes in the means of mutual reciprocity (level 2), which changed from 10.6 to 10.0 (*p* = 0.33), nor in mutual cooperation (level 3), which changed from 10.2 to 9.1 (*p* = 0.12).

### 3.4. SFAB

Interpersonal ability was examined based on the adaptation in the social aspect of the intellectually disabled, and a significant improvement was observed at the physical level (level 0, *p* = 0.02) and unilateral level (level 1, *p* = 0.01). The HT program significantly improved the physical and vocational behaviors of persons with intellectual disabilities from 29.9 to 38.6 and from 25.4 to 25.7, respectively, although there was no statistically significant difference (Table 6).

### 3.5. Horticultural Job Evaluation

The results of the subcategory-wise analysis of horticultural job evaluations are shown in Table 7. The HT program significantly improved: social competence by 4 points, from 17.2 to 21.2 (*p* = 0.02); practical skills competency by 1.4 points, from 10.1 to 11.5 (*p* = 0.02); and mental and physical competence by 1.1 points, from 9.7 to 10.8 (*p* = 0.02).

### 3.6. Changes in Neurotrophic Factors

Blood samples before and after the program were tested for brain nerve growth factors, such as BDNF, VEGF, and IGF-1. The BDNF and IGF-1 levels increased from 7.702 pg/mL to 7.833 pg/mL and 8.151 pg/mL to 8.215 pg/mL, respectively; there was no statistically significant difference. However, the VEGF increased by 6.8%, from 615.3 pg/mL to 657.4 pg/mL, showing a positive effect (*p* = 0.05) (Table 8).

### 3.7. Satisfaction with the 12-Session Horticultural Therapy Program

The participants completed a satisfaction survey at the end of the HT program. Regarding the overall interest in the program, 92.8% (13 participants) were very interested, and 7.1% (1 participant) were interested. Regarding the satisfaction with the duration of the sessions, 71.4% (10 participants) were very satisfied, 21.4% (3 participants) were satisfied, and 7.1% (1 participant) were neutral. Regarding the difficulty of the program, 85.7% (12 participants) answered “easy to understand”, and 14.2% (2) answered “neutral”. In addition, 85.7% (12 participants) answered that they would like the program to continue.

In the HT program, planting and decoration (20.5%) was the most common preference, followed by the arrangement of flower baskets (17.9%), sowing (15.4%), planting garden crops (10.3%), and making succulent art (10.3%). This was followed by sprouting (7.7%); hydroponics, ornamental plants, and basket harvesting (5.1%); and leafy vegetable sandwich preparation and herb planting (2.6%).

## 4. Discussion

The purpose of this study was to investigate the effects of the 12-session HT on improvements in work performance and interpersonal relationships among persons with intellectual disabilities in the protected workplace.

Prior to conducting the study, researchers observed that persons with intellectual disabilities in a protective workplace mainly used their upper limbs and hands while performing their tasks. Since the HT program consisted of repeated planting and sowing, step by step, to improve hand functions and upper limb movements during horticultural activities, it improved handgrip power (*p* = 0.05), hand strength and endurance (*p* = 0.04), and hand agility (*p* = 0.02). Our results were consistent with those of previous studies since they showed that the HT program, comprising simple to delicate work, improved the grip power of both hands [27], and HT improved the pinch and grip of the elderly with dementia [28]. In addition, previous studies have shown that the hand agility of persons with intellectual disabilities increased after performing horticultural activities such as planting, topiary, and crafts, including making pressed flowers [29]. In addition, Kim, Park, and Kim [30] suggested that various gardening activities, such as cultivating crops with both hands in healing gardens, improved hand functions, especially hand agility.

To improve the hand functions of persons with intellectual disabilities in the protective workplace, we classified the size of seeds as large, medium, and small to gradually increase the hand agility. For watering, we asked the participants to use sprayers to spray seedlings five times each, alternately with their right and left hands. Watering was performed daily to increase the grip and improve the function of both hands [20]. The program consisted of garden activities such as planting seedlings, managing gardens, and watering that exercised the lower limbs [20] and used the grip of hands [31]. Herb and succulent cuttings demonstrated the physical effects of improving the speed of work and improving small muscles, since knowing which tools were to be used and which parts of the plant were to be cut was important. After the program, the mean number of completions in the bag strapping task increased significantly from 9.9 bags to 11.3 bags after the program (*p* = 0.00) (Table 4).

Although research targets were different, the results of this study were in line with the results of previous studies that found that the elderly who have performed horticultural activities showed significantly higher hand functions (hold, finger strength) than those who did not [31], and that flower arrangement work in horticultural therapy programs has a positive effect on hand and upper limb function improvement in stroke patients [32]. Flower arranging in the horticultural treatment program has a positive effect on the improvement of hand and upper extremity function in stroke patients [32], and an electromyography test showed that 15 common horticultural activities affect specific upper extremity and hand muscle activation [16]. In addition, as a result of performing horticultural activities on students with intellectual disabilities, a medium-intensity physical activity that used both the upper and lower bodies at the same time was effective in improving hand functions [23]. This study also showed effects in hand function and physical activity.

The present study showed significant increases in the impulse-physical level (level 0) and unilateral level (level 1) (mean changed from 14.1 to 15.7; *p* = 0.01). During the horticultural activities, participants were asked to form groups and select their group leaders. Through this process, they were able to communicate with each other and take responsibility for garden management. The cutting activity helped them understand how to control their feelings. This implies that intellectually impaired persons with strong impulses and a lack of understanding of others may be able to alleviate impulses and improve their understanding of others through gardening activities. These results were the same as those in studies that showed decreases in hyperactivity and aggression along with a reduction in antisocial behavior and rebellion in intellectually or mentally impaired students [33,34,35]. However, there were no significant changes in levels 2 and 3. This result may have been influenced by the short duration of the program, being only six weeks. To recognize and understand others, longer horticultural programs should be conducted in future studies.

A horticultural job evaluation is a self-reported questionnaire that measures a person’s horticultural activity skills through simple questions that persons with intellectual disabilities can understand. This assessment evaluates the understanding of gardening skills, fulfillment of responsibilities, and physical care. The present program significantly improved all three subcategories in the horticultural job evaluation. Horticultural activity is an activity that anyone can do and is known to be particularly suitable for persons with intellectual disabilities since the repetitive and continuous gardening activities could enhance their adaptability. The activities could help them develop a sense of responsibility and achievement through watering, weeding, harvesting, and managing gardens. Community gardening could help them become more considerate and understand others. Furthermore, physical activity of appropriate intensity and horticultural activity that uses the upper and lower bodies may suitably affect their mental health. Persons with intellectual disabilities can experience positive physical and mental changes, such as stress relief, through breathing fresh air, cultivating various plants, and cooking from the harvest.

VEGF is an important neurotrophic factor that regulates vasculogenesis in embryo development, in addition to angiogenesis in vivo, and is a key factor in regulating the production and plasticity of neurons. In particular, it plays a vital role in regulating functions and explaining the hippocampus’s structural changes [36]. Exercise has been reported as a representative factor that improves the function of the hippocampus by controlling functional changes such as neuroplasticity by regulating brain nerve growth factors, such as NGF and VEGF, and structural changes, such as neuronal cell formation [37]. A study was conducted on the effect of 12 weeks of regular exercise on vascular structure, function, and VEGF in obese middle-aged men. As a result, regular aerobic exercise has been shown to improve abdominal fat and serum lipid/lipoprotein, decrease carotid peak systolic and diastolic blood flow rates, and increase VEGF, an angiogenic factor [38]. As a result of the effect of short-term gardening activities on cognitive function-related brain nerve growth factors in the elderly, BDNF significantly increased, but VEGF did not improve to the same degree [39]. This is thought to be related to the positive effect of regular aerobic exercise for 12 weeks or longer on the improvement of cognitive function, NGF, and VEGF rather than short-term [40].

However, in this study, it is thought that regular physical activity for 12 sessions had a positive effect on VEGF related to cognitive improvement. The results of this study also showed a tendency to increase VEGF, suggesting that HT may have a positive effect on brain nerve growth factors of persons with intellectual disabilities. Horticultural activity is a low-to-moderate-intensity physical activity [41], similar to that of aerobic exercises. Therefore, it is thought to have the possibility of positively affecting cognition by controlling the function of the hippocampus.

The limitations of this study are that the control group could not be formed due to practical difficulties, only 14 subjects were surveyed, and the program period was short. These limitations are closely related to the fact that the subjects of the study were the intellectually disabled. Therefore, the results of this study need to be interpreted carefully.

This study was conducted on the intellectually disabled who were engaged in making paper bags in the sheltered workshop. Further studies will be able to examine whether horticultural therapy activities have a positive effect on improving work performance and interpersonal relationships in more diverse work areas (e.g., electronic component assembly) of people with intellectual disabilities working in a sheltered workshop. Furthermore, more research will be needed to verify the effect of changes in brain nerve growth factors related to cognitive function analyzed in this study. Based on the results, it will be possible to find a way to connect the HT program to the vocational rehabilitation program for the intellectually disabled.

## 5. Conclusions

Occupational rehabilitation is the most important cornerstone for community therapy because it helps the intellectually disabled return to society when applied to their abilities [42]. The researchers aimed to develop a HT program that would improve the work performance of the participants by analyzing their work in a protective workplace. Consequently, it positively affected vocational rehabilitation for persons with intellectual disabilities. In particular, it presented the effectiveness of low-to-moderate-intensity horticultural activities using the upper body and hands. Moreover, this study aimed to scientifically verify the physical and cognitive effects of horticultural activity by conducting neurotrophic factor-related tests for persons with intellectual disabilities.

It was difficult to recruit a control group because of the characteristics of persons with intellectual disabilities. Another limitation was the difficulty in clarifying the effects of previous studies in a short period of six weeks. To complement this study, further studies with larger sample sizes are needed.

In conclusion, if the work of the intellectually disabled is analyzed and applied to a scientific horticultural therapy program, social factors such as interpersonal relationships and hand functions could be improved to provide positive possibilities for increasing work efficiency. In addition, this study found changes in the brain nerve growth factor related to cognitive function before and after participating in the program, so that additional research on the effect of horticultural therapy on cognitive changes is needed.

## Figures and Tables

**Table 1:** Demographic characteristics of the participants (*n* = 14).

Item		Mean ± Standard Deviation
Age		33.9 ± 9.4
Number of years worked		7.3 ± 4.5
Body mass index (kg/m^2^)		26.4 ± 4.8
		% (*n*)
Gender	Male	43.0 (6)
	Female	57.0 (8)
Level of disability	Grade 1 (IQ 50~69)	14.3 (2)
	Grade 2 (IQ 35~49)	57.1 (8)
	Grade 3 (IQ 20~34)	28.6 (4)

**Table 2:** A 12-session horticultural therapy program for improving the work performance and interpersonal relationships of persons with intellectual disabilities.

Session	Horticultural Activity	Physical Activity	
		Upper Limb Functions and Hands	Lower Limb Functions
1	Spring! go start	Sowing seeds	Making garden plots, fertilizing
2	Planting plants	Planting plants	Watering
3	Sowing seeds	1-step sowing large seeds, spraying	Watering
4	Sowing vegetable sprouts	2-step sowing medium seeds, spraying	Watering
5	Cutting herbs and transplanting	Cutting, spraying	Weeding
6	Making sandwiches	Holding	Harvest holding
7	Succulent art	Cutting, spraying	Watering
8	Harvest basket	Packing the harvest	Weeding, harvest
9	Topiary	Topiary, watering	Weeding
10	Hydroponics	3-step sowing small seeds, spraying	Watering
11	Decorating a flowerpot	Planting plants, spraying	Watering
12	A flower basket	Planting plants	

**Table 3:** Hand function tests for the entire group before and after the 12-session horticultural therapy.

Item		Mean (Standard Deviation)		
	Pre-Test	Post-Test	*Z*	*p*-Value
Pegboard—fingertips	146.3 (54.4)	121.7 (44.2)	−1.925 ^b^	0.05 (NS)
Pinch gauge—gripping	18.6 (6.9)	20.8 (8.1)	−2.103	0.04 *
Fingertips—grip power	6.5 (2.9)	7.4 (2.5)	−2.359 ^b^	0.02 *

* *p* < 0.05; NS = non-significant; ^b^. based on the positive rank.

**Table 4:** Results of individual work activities (number of bag straps) before and after the 12-session horticultural therapy.

Item		Mean (Standard Deviation)		
	Pre-Test	Post-Test	*Z*	*p*-Value
Bag straps	9.9 (2.6)	11.3 (2.7)	−2.701 ^b^	0.00 **

** *p* < 0.01; ^b^. based on the positive rank

**Table 5:** Changes in scores based on subareas of interpersonal relationships before and after the 12-session horticultural therapy.

Item		Mean (Standard Deviation)		
	Pre-Test	Post-Test	*Z*	*p*-Value
Level 0	14.4 (3.1)	15.2 (2.9)	−2.326 ^b^	0.02 *
Level 1	14.1 (3.5)	15.7 (2.4)	−2.501 ^b^	0.01 *
Level 2	10.6 (4.1)	10.0 (4.1)	−0.957 ^c^	0.33
Level 3	10.2 (4.0)	9.1 (3.9)	−1.550 ^c^	0.12
Total	49.4 (10.2)	50 (9.9)	−0.845 ^b^	0.40

* *p* < 0.05; level 0: impulse-physical; level 1: unilateral level; level 2: mutual reciprocity; level 3: mutual cooperation.; ^b^. based on negative ranking; ^c^. based on the positive rank.

**Table 6:** Changes in the vocational skill and physical ability scores based on the entire group’s subareas before and after the 12-session horticultural therapy.

Item			Mean (Standard Deviation)		
		Pre-Test	Post-Test	*Z*	*p*-Value
Vocational skill	Body ability	29.9 (7.6)	38.6 (9.7)	−3.235 ^b^	0.00 **
Physical ability	Vocational behavior	25.4 (8.3)	25.7 (8.8)	−1.267 ^b^	0.20

** *p* < 0.01; ^b^. based on the positive rank.

**Table 7:** Changes in the horticultural job evaluation results of participants before and after the 12-session horticultural therapy.

Item		Mean (Standard Deviation)		
	Pre-Test	Post-Test	*Z*	*p*-Value
Social competence	17.2 (5.5)	21.2 (13.2)	−2.410 ^b^	0.02 *
Job competence	10.1 (2.9)	11.5 (2.3)	−2.401 ^b^	0.02 *
Mental health competence	9.7 (3.3)	10.8 (2.7)	−2.401 ^b^	0.02 *

* *p* < 0.05; ^b^. based on the positive rank.

**Table 8:** Brain nerve growth factor changes before and after the 12-session horticultural therapy.

Item		Mean (Standard Deviation)		
	Pre-Test	Post-Test	*Z*	*p*-Value ^1^
BDNF ^1^ (pg/mL) ^2^	7.702 (0.761)	7.833 (0.713)	−0.534 ^b^	0.594
VEGF (pg/mL) ^3^	615.3 (138.0)	657.4 (166.8)	−1.947 ^b^	0.052
IGF-1 (ng/mL) ^4^	17.5 (22.76)	17.0(23.38)	−0.31 ^b^	0.975

^1^*p* < 0.05; ^b^. based on the positive rank. ^2^ BDNF is brain-derived neurotrophic factor. ^3^ VEGF is vascular endothelial growth factor. ^4^ IGF-1 is insulin-like growth factor 1.

## Data Availability

The datasets generated for this study are available on request to the corresponding author.

## References

[1] (2022). Korea Statistical Information Service. https://kosis.kr/statHtml/statHtml.do?orgId=117&tblId=DT_11761_N003.

[2] Cho M.N. (2009). A Study on the Job Rehabilitation Program of Hearing-Impaired People: Through Flower Offering of the Sutra and Horticultural Therapy. Master’s Thesis.

[3] Auerbach E.S., Richardson P. (2005). The long-term work experiences of persons with severe and persistent mental illness. Psychiatr. Rehabil. J..

[4] McGurk S.R., Mueser K.T., Buckley P.F., Gaughran F. (2004). Vocational rehabilitation for severe mental illness. Treatment-Refractory Schizophrenia.

[5] Blustein D.L. (2006). The Psychology of Working: A New Perspective for Career Development, Counseling, and Public Policy.

[6] Lee H.J. (2010). A Study on Parent’s Awareness and Desire on Vocation Rehabilitation Service for the Mentally Retarded. Master’s Thesis.

[7] Chang B.H. (2005). A Study of vocational adaptive skills required to the adolescents with mental retardation in employment-site. J. Vocat. Rehabil..

[8] Emener W.G. (1980). Relationships among rehabilitation counselor characteristics and rehabilitation client outcomes. Rehabil. Couns. Bull..

[9] Son K., Jung S., Lee A., Park S. (2016). The theoretical model and universal definition of horticultural therapy. Acta Hortic..

[10] Relf P.D., Lohr V.I. (2003). Human Issues in Horticulture. HortScience.

[11] Son K.C., Cho M.G., Song J.O., Kim S.Y., Lee S.S. (2006). Practice of Professional Horticultural Therapy.

[12] Park S.-A., Lee A.-Y., Son K.-C., Lee W., Kim D. (2016). Gardening Intervention for Physical and Psychological Health Benefits in Elderly Women at Community Centers. HortTechnology.

[13] Park S.-A., Shoemaker C., Haub M. (2008). Can Older Gardeners Meet the Physical Activity Recommendation through Gardening?. HortTechnology.

[14] Park H.-J., Oh D.-W., Kim S.-Y., Choi J.-D. (2011). Effectiveness of community-based ambulation training for walking function of post-stroke hemiparesis: A randomized controlled pilot trial. Clin. Rehabilitation.

[15] Park S.-A., Lee H.-S., Lee K.-S., Son K.-C., Shoemaker C.A. (2013). The Metabolic Costs of Gardening Tasks in Children. HortTechnology.

[16] Park S.-A., Oh S.-R., Lee K.-S., Son K.-C. (2013). Electromyographic Analysis of Upper Limb and Hand Muscles during Horticultural Activity Motions. HortTechnology.

[17] Park S.-A., Lee A.-Y., Kim J.-J., Lee K.-S., So J.-M., Son K.-C. (2014). Electromyographic Analysis of Upper and Lower Limb Muscles during Gardening Tasks. Korean J. Hortic. Sci. Technol..

[18] Lee S.J. (2013). The Development and Application of Horticultural Therapy Introduced Learning Theory of Constructivism—Focused on Vocational Training for Adolescents with Intellectual Disabilities. Ph.D. Thesis.

[19] Park H.M., Kim G.S., Lee O.S., Song C.E. (2010). Effect of Horticultural Therapy Program on the Vocational Rehabilitation in a Mentally Retarded Person. Korean Inst. For. Recreat. Welf..

[20] Lee A.Y. (2017). Analysis of Kinematic and Kinetic Characteristics of Horticultural Therapy Activity and Verification of Its Rehabilitative Effectiveness. Ph.D. Thesis.

[21] Nam O.C., Goo J.H., Son K.C. (1999). A Vocational rehabilitation model for the mentally retarded: The Eun-Hyang horticultural therapy and training program. Korean J. Hortic. Sci. Technol..

[22] Hannania R., Smith L.-M. (2010). Selective attention and attention switching: Towards a unified developmental approach. Dev. Sci..

[23] Joo B.-S., Park S.-A., Son K.-C. (2012). Improving Work Adjustment Skills in Students with Mental Retardation Using Hydroponics Program. Korean J. Hortic. Sci. Technol..

[24] Ruff R.M., Parker S.B. (1993). Gender- and Age-Specific Changes in Motor Speed and Eye-Hand Coordination in Adults: Normative Values for the Finger Tapping and Grooved Pegboard Tests. Percept. Mot. Ski..

[25] Kim N.E. (2004). Development of Horticultural Activity Performance Assessment (HAPA) for Mild and Moderate Mentally Retarded People. Korean J. Hortic. Sci. Technol..

[26] Kim S.Y. (2018). Development of Competency-Based Horticulture Rehabilitation Assistant Curriculum for Developmental Disability Students. Master’s Thesis.

[27] Ahn J.Y. (2007). Effect of Horticultural Therapy on Hand Function and Quality of Life of the Crippled Disordered Persons in the Group Home. Master’s Thesis.

[28] Yun S.Y. (2007). Effects of the Application of Horticultural Therapy Based on Pincus ‘Theory of Rehabilitation Practice on Demented Elders’ Daily Activity Functions and Physiological Changes. Ph.D. Thesis.

[29] Lee M.H. (2008). Effects of Horticultural Therapy on the Self-esteem, Psychosocial and Emotional Behavior, and Hand Function in Disabled Person. Master’s Thesis.

[30] Kim M.-Y., Park W.-K., Kim S.-Y. (2016). Effect of Horticultural Occupation Therapy Program Utilizing Healing Garden on Emotion Stability and Hand Dexterity of Chronic Mental Disorder Patients. J. Korean Soc. People Plants Environ..

[31] Park S.-A., Shoemaker C.A., Haub M.D. (2009). Physical and Psychological Health Conditions of Older Adults Classified as Gardeners or Nongardeners. HortScience.

[32] Lee S.-S., Park S.-A., Kwon O.-Y., Song J.-E., Son K.-C. (2012). Measuring Range of Motion and Muscle Activation of Flower Arrangement Tasks and Application for Improving Upper Limb Function. Korean J. Hortic. Sci. Technol..

[33] Bang G.H., Kim J.H. (2004). The Effect of Horticultural Therapy on the Maladjusted Behavior of Children with Mental Retardation. J. Agric. Educ. Hum. Resour. Dev..

[34] Lee M.S., Kim H.Y., Shin J.E., Ku J.H. (2008). Effects of Horticultural Therapy on The Adaption Behavior and Durability of student in special school. J. People Plants Environ..

[35] Shim Y.E. (2008). Effect of Horticultural Therapy Program for Improvement of Work Adjustment Skill in People with Mental Retardation. Master’s Thesis.

[36] Lanni C., Stanga S., Racchi M., Govoni S. (2010). The Expanding Universe of Neurotrophic Factors: Therapeutic Potential in Aging and Age-Associated Disorders. Curr. Pharm. Des..

[37] Adlard P.A., Perreau V.M., Cotman C.W. (2005). The exercise-induced expression of BDNF within the hippocampus varies across life-span. Neurobiol. Aging.

[38] Park S.K., Kwon Y.C., Park J.K. (2008). Effect of aerobic exercise on carotid artery structure, function and vascular endothelial growth factor(VEGF) in abdominal obese men. Korean J. Phys. Educ..

[39] Park S.-A., Lee A.-Y., Park H.-G., Lee W.-L. (2019). Benefits of Gardening Activities for Cognitive Function According to Measurement of Brain Nerve Growth Factor Levels. Int. J. Environ. Res. Public Health.

[40] Kramer A.F., Colcombe S.J., McAuley E., Scalf P.E., Erickson K.I. (2005). Fitness, aging and neurocognitive function. Neurobiol. Aging.

[41] Park S.-A., Lee A.-Y., Son K.C. (2015). A Comparison of Exercise Intensity Between Two Horticultural and Four Common Physical Activities Among Male Adults in Their 20s. Hortic. Sci. Technol..

[42] Lamp H.R., Lamp H.R. (1976). Guiding Principle for Community Survival, in Community Survival for Long-Term Patients.

